# Field evaluation of a simple and rapid diagnostic test, RLDT to detect *Shigella* and enterotoxigenic *E. coli* in Indian children

**DOI:** 10.1038/s41598-024-59181-6

**Published:** 2024-04-16

**Authors:** Goutam Chowdhury, Debjani Ghosh, Yiyi Zhou, Alok K. Deb, Asish Kumar Mukhopadhyay, Shanta Dutta, Subhra Chakraborty

**Affiliations:** 1 Division of Bacteriology, ICMR-National Institute of Cholera and Enteric Diseases, P33 CIT Road, Scheme XM, Beliaghata, Kolkata 700010 India; 2 Department of International Health, Johns Hopkins Bloomberg School of Public Health, Baltimore, MD USA

**Keywords:** ETEC, Shigella, Diarrhea, Diagnostics, RLDT, qPCR, Culture, Genetic techniques, Epidemiology, Paediatric research, Biological techniques

## Abstract

The diagnostic assays currently used to detect *Shigella* spp*.* (Shigella) and enterotoxigenic *Escherichia coli* (ETEC) are complex or elaborate which make them difficult to apply in resource poor settings where these diseases are endemic. The simple and rapid nucleic acid amplification-based assay "Rapid LAMP-based Diagnostic Test (RLDT)" was evaluated to detect *Shigella spp* (Shigella) and enterotoxigenic *Escherichia coli* (ETEC) and determine the epidemiology of these pathogens in Kolkata, India. Stool samples (n = 405) from children under five years old with diarrhea seeking care at the hospitals were tested, and 85(21%) and 68(17%) by RLDT, 91(23%) and 58(14%) by quantitative PCR (qPCR) and 35(9%) and 15(4%) by culture, were positive for Shigella and ETEC, respectively. The RLDT showed almost perfect agreement with qPCR, Kappa 0.96 and 0.89; sensitivity 93% and 98%; specificity 100% and 97% for Shigella and ETEC, respectively. While RLDT detected additional 12% Shigella and 13% ETEC than culture, all culture positives for Shigella and ETEC except one each were also positive by the RLDT, sensitivity 97% and 93% respectively. RLDT is a simple, sensitive, and rapid assay that could be implemented with minimum training in the endemic regions to strengthen the disease surveillance system and rapid outbreak detection.

## Introduction

*Shigella* spp. (Shigella) and enterotoxigenic *Escherichia coli* (ETEC) are the leading enteropathogens causing significant diarrheal morbidity and mortality in children less than 5 years of age in sub-Saharan Africa and South Asian regions and could cause adverse long-term consequences including childhood stunting^1^. Recent longitudinal studies have found that Shigella and ETEC are the most common organisms requiring targeted interventions^2^. In the global burden study, Shigella and ETEC together were responsible for more than 427,000 deaths in 2015 which ranked second and fourth respectively regarding pathogen contributions to global diarrheal deaths^1^. Licensed vaccines are not yet available for either pathogen, but vaccine candidates are under development^3^.

The genus *Shigella* includes four species, namely, *S. flexneri, S. dysenteriae, S. boydii,* and *S. sonnei*, and each of these species is further classified into 6, 15, 23, and 1 serogroup respectively, based on the ‘O’ antigen component of the lipopolysaccharide^4,5^. Among the four species, currently, *S. flexneri* and *S. sonnei* are the major pathogens frequently reported in low and middle-income countries^1,6^. The severity of shigellosis is dependent on the various virulence factors located in the chromosome or large virulent *inv* plasmids. The invasion plasmid antigen H (*ipaH*) gene is common in all the *Shigella* serogroups and serotypes and is present in multiple copies located on both the plasmid and chromosome. The *ipaH* gene is responsible for the diffusion of Shigella in epithelial cells^7^. The *ipaH* gene is also found in enteroinvasive *E. coli* (EIEC). ETEC colonizes the surface of the small intestine using the colonization factor antigens and elaborate heat-labile toxins (LT) and/or heat-stable toxins (STh or STp) which leads to secretory diarrhea^8,9^.

While culture remained the gold standard for the detection of Shigella, PCR to detect the toxin genes from the *E. coli* isolates is the most frequently used technique to detect ETEC. Shigella culture is a time-consuming, laborious activity that requires multiple subcultures, biochemical and serological confirmation, and is not a sensitive diagnostic assay^10,11^. Molecular technologies, such as PCR and quantitative PCR (qPCR) assays performed from purified DNA are highly sensitive; however, they are often not available in the endemic regions because of the requirement of specialized laboratories, trained personnel, expensive reagents and equipment, and the risk of contamination^12,13^.

Chakraborty et al*.* have developed a molecular diagnostic assay, Rapid LAMP-based Diagnostic Test (RLDT), for the detection of ETEC and Shigella directly from stool specimens^14^. RLDT is a simple, rapid, sensitive, and low-cost, cold chain and mostly electricity-free nucleic acid amplification-based method.

In this study, we evaluated the performance of the RLDT compared to qPCR and culture methods using stool specimens collected from the children with diarrhea and determined the feasibility of implementation of the RLDT in an ETEC and Shigella endemic setting, in Kolkata, India. We also determined the age-specific prevalence and clinical outcomes of Shigella and ETEC diarrhea in the study area.

## Results

Comparative analysis between RLDT, qPCR, and culture for detection of Shigella and ETEC. Overall, 405 stool samples were tested by the RLDT, qPCR, and culture for Shigella and ETEC. RLDT was done directly from the stool using the RLDT kit while qPCR was done from the purified DNA from the stool.

### Shigella

**Figure 1:**
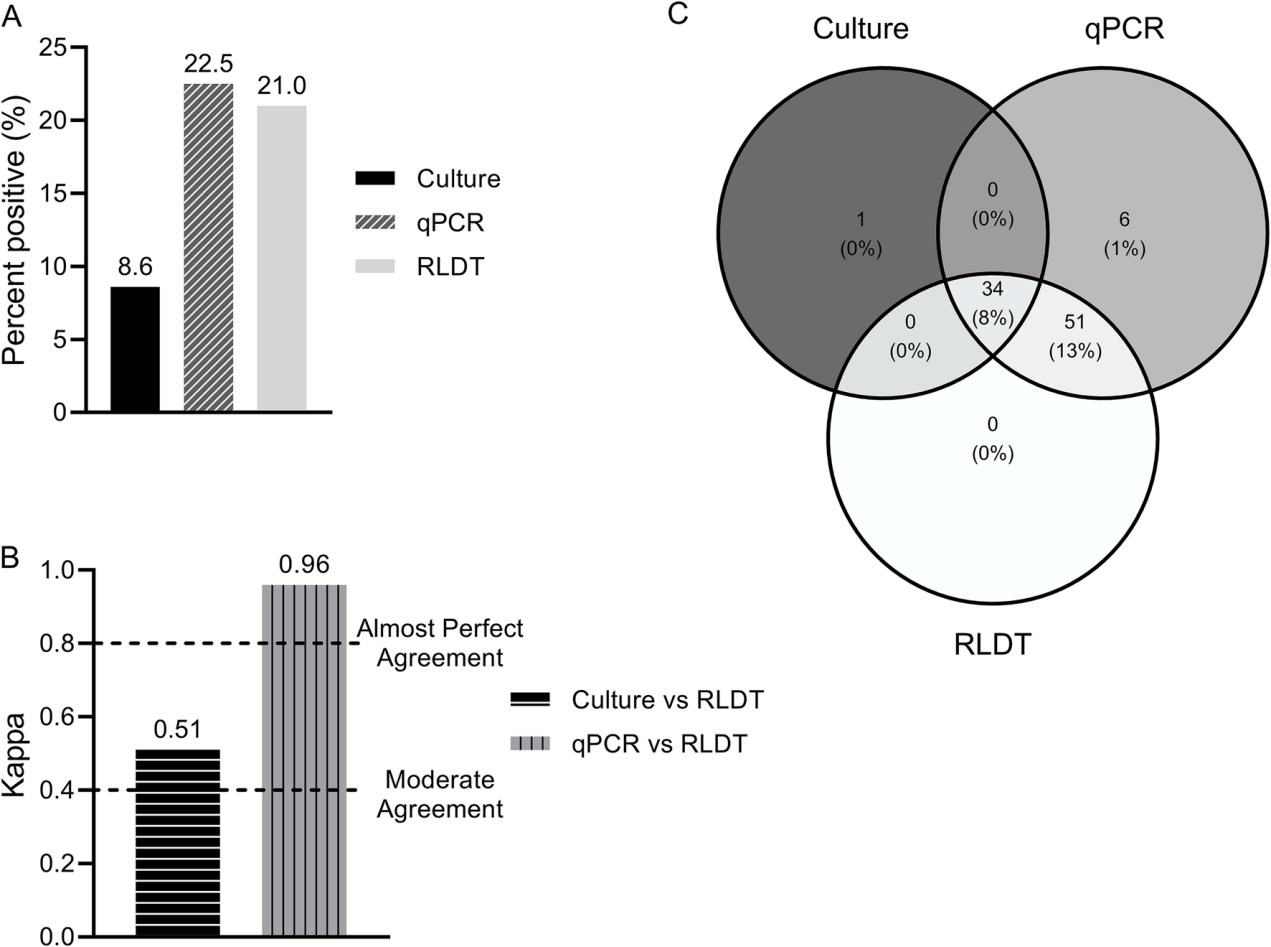
Comparison between the RLDT, qPCR, and culture in the detection of Shigella. Figure 1(**a**). Percent positives of Shigella by culture, qPCR, and RLDT; Fig. 1(**b**). Agreement between the RLDT and culture and RLDT and qPCR using Cohen’s kappa; Fig. 1(**c**). Venn diagram depicting identification overlap between the three assays.

Out of 405 samples tested for Shigella, 22.5% (91 of 405) were positive by the qPCR, 21% (85 of 405) by the RLDT, and 8.6% (35 of 405) by the culture (Fig. 1a). The sensitivity and specificity of RLDT for Shigella compared to qPCR were 93.4% and 100% respectively and compared to culture were 97.1% and 86.2% respectively (Table 1). The positive and negative predictive values (PPV and NPV) and accuracy values are listed in Table 1.

**Table 1:** Sensitivity and specificity of RLDT comparing with qPCR and Culture in India.

Targets	Total samples screened	Prevalence by RLDT (%)	Prevalence by qPCR or culture (%)	False positive	False negative	Sensitivity (%)	Specificity (%)	PPV (%)	NPV (%)	Accuracy (%)
Shigella										
QPCR vs RLDT										
ipaH	405	85 (21.0)	91 (22.5)	0	6	93.4	100.0	100.0	98.1	98.5
Culture vs RLDT										
ipaH	405	85(21.0)	35(8.6)	51	1	97.1	86.2	40.0	99.7	87.2
ETEC										
QPCR vs RLDT										
ETEC total	404	68 (16.8)	58 (14.4)	11	1	98.3	96.8	83.8	99.7	97.0
LT	404	47 (11.6)	43 (10.6)	6	2	95.3	98.3	87.2	99.4	98.0
STh	404	22 (5.4)	24 (5.9)	0	2	91.7	100.0	100.0	99.5	99.5
STp	404	26 (6.4)	18 (4.5)	8	0	100.0	97.9	69.2	100	98.0
Culture vs RLDT										
ETEC total	405	68 (16.8)	15 (3.7)	54	1	93.3	86.2	20.6	99.7	86.4
ETEC LT	405	47 (11.6)	8 (2.0)	39	0	100.0	90.2	17.0	100.0	90.4
ETEC ST	405	44 (10.9)	12 (3.0)	33	1	91.7	91.6	25	99.7	91.6

To calculate the agreement between paired assays, Cohen’s Kappa statistics were used. For the detection of Shigella, qPCR vs RLDT showed almost perfect agreement (0.96) while culture vs RLDT showed moderate agreement (0.51) since culture is less sensitive (Fig. 1b).

Based on the Venn diagram tool (Fig. 1c) 34 samples were positive for Shigella by all the 3 assays, culture, qPCR, and RLDT. Six samples were only positive by the qPCR, of which five samples had a Cq value close to the threshold (Cq29 to 31). With changing the cut-off to Cq28 (CFU ~ 10^5^ CFU/gm of stool), the sensitivity of RLDT was increased to 98.8%. RLDT and qPCR detected 51 (13%) more samples that were negative by culture. All the culture positives were also positive by the RLDT except one sample. This sample was culture positive for *S. Sonnei,* but negative by RLDT and qPCR with the Cq cut-off used.

### ETEC

**Figure 2:**
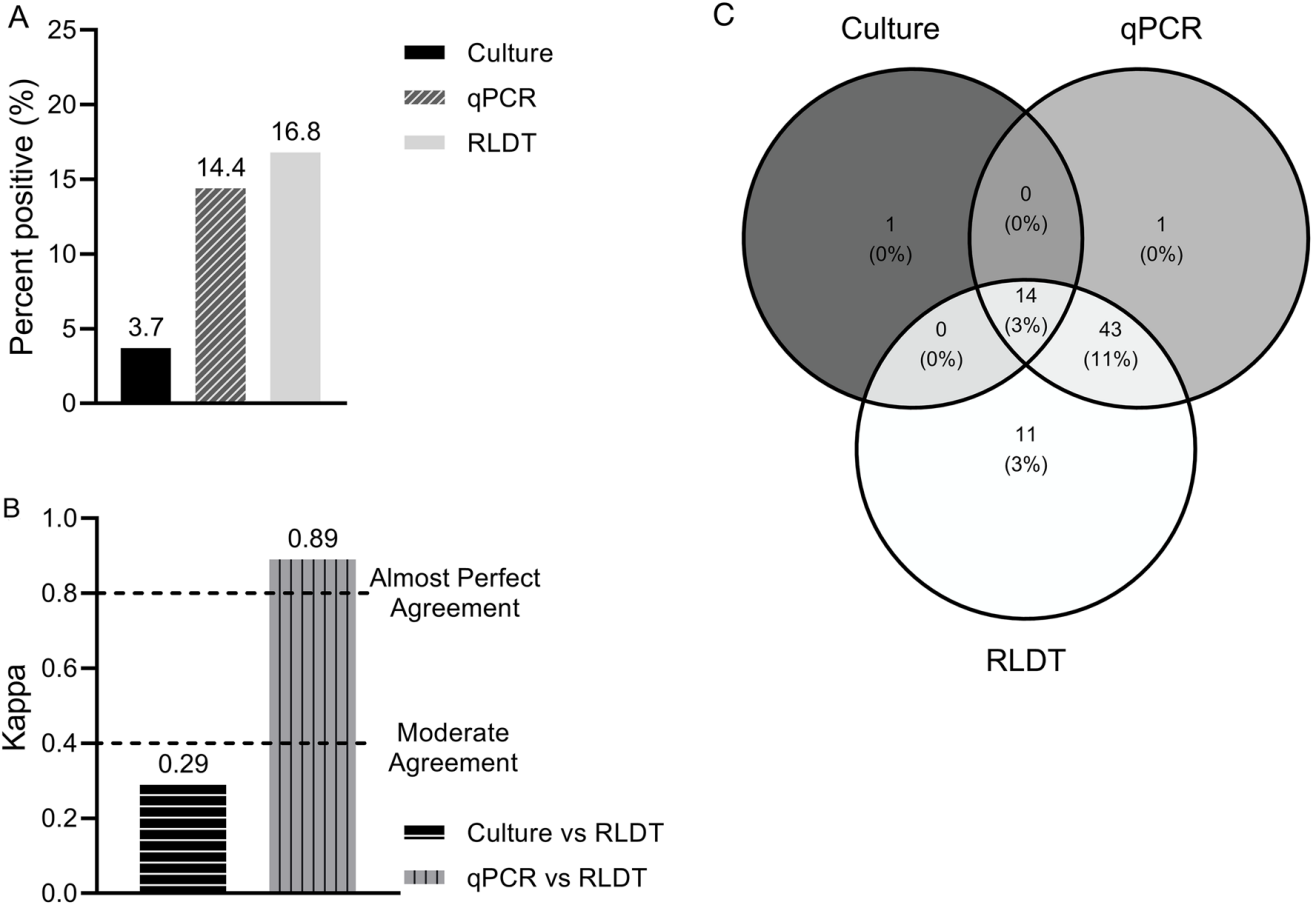
Comparison between the RLDT, qPCR, and culture in the detection of ETEC. Figure 2(**a**). Percent positives of ETEC by culture, qPCR, and RLDT. A sample is positive for ETEC when at least one of the genes LT, STh, or STp is positive. Figure 2(**b**). Agreement of ETEC between the RLDT and culture and RLDT and qPCR using Cohen’s kappa; Fig. 2(**c**). Venn Diagram depicting identification of ETEC overlap between the three assays.

Among the stool specimens tested for ETEC 16.8% (68 of 405) were positive by the RLDT, 14.4% (58 of 404) by qPCR, and only 3.7% (15 of 405) were positive by culture followed by conventional PCR of the *E. coli* isolates (Fig. 2a). The qPCR for ETEC genes were performed on 404 stool samples since there was not enough sample volume and extracted DNA for one stool sample.

Overall, the sensitivity and specificity of RLDT in the detection of ETEC compared to qPCR were 98.3% and 96.8% respectively (Table 1). The PPV, NPV and accuracy values are listed in Table 1. The sensitivity of the RLDT compared to qPCR was high for STp (100%), followed by LT (95.3%) and STh (91.7%). The specificity for STh was 100% followed by LT (98.3%) and STp (97.9%). The sensitivity and specificity of RLDT compared to culture were 93.3% and 86.2% respectively. Among the ETEC genes, LT showed the highest sensitivity, 100% followed by ST 91.7% compared to culture. The specificity was high for ST (91.6%) followed by LT (90.2%).

The agreement between paired assays, qPCR, and RLDT had almost perfect agreement (0.89) while culture vs RLDT resulted in less than moderate agreement (0.29) as the culture is less sensitive (Fig. 2b).

Based on the Venn diagram (Fig. 2c), 14 samples were positive by all three assays. The qPCR and RLDT detected 43 (11%) more ETEC positive samples than culture. RLDT detected 11 more samples than qPCR. Since these samples were not further tested by another assay of similar sensitivity, it was not possible to confirm if these were false or real positives.

**Figure 3:**
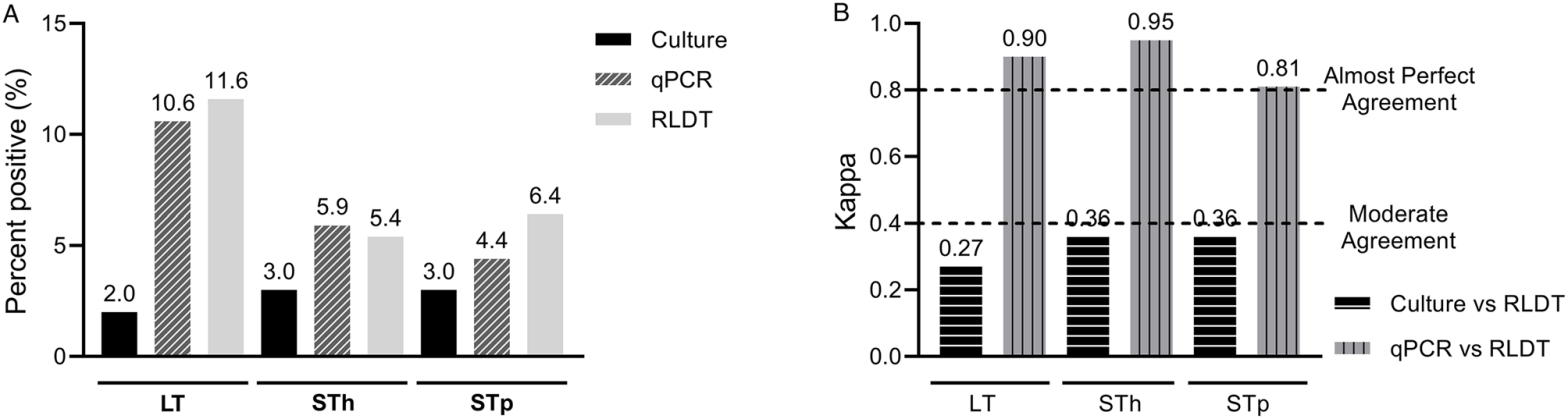
Comparison between the RLDT, qPCR, and culture among the ETEC toxin genes. Figure 3(**a**). Percent positives of LT, STh, or STp by culture, qPCR, and RLDT. The culture was done for ST, not differentiated by the ST types STh and STp; Fig. 2b. Agreement of LT, STh, or STp between the RLDT and culture and RLDT and qPCR using Cohen’s kappa.

Among the ETEC toxin genes, the positivity rates by RLDT were 11.6% for LT, 5.4% for STh, and 6.4% for the STp gene. Using qPCR, the positivity rates were 10.6% for LT, 5.9% for STh, and 4.4% for STp gene. Culture could detect LT in 2% (8) and ST in 3% (12) of the samples (Fig. 3a). Among ETEC, LT showed the highest sensitivity, 100% followed by ST 91.7% compared to culture. The specificity was high for ST (91.6%) followed by LT (90.2%) (Table 1). The overall performance of ETEC toxin genes, qPCR vs RLDT showed almost perfect agreement (0.81 to 0.95) while the agreement of culture vs RLDT remained low (0.27 to 0.36) (Fig. 3b).

Sixteen samples were positive for both ETEC and Shigella by qPCR and RLDT.

### Prevalence of Shigella and ETEC and clinical severity

**Figure 4:**
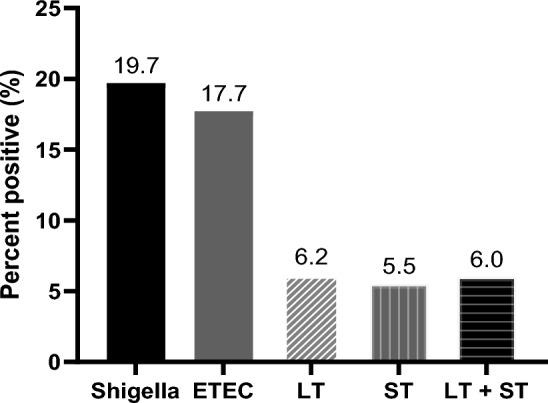
Prevalence of Shigella and ETEC in Kolkata, India by RLDT. Proportion of the children with diarrhea seeking care at the study hospitals, positive for Shigella and ETEC and ETEC toxin types (LT, ST, and LT + ST) by RLDT.

The prevalence of Shigella and ETEC in the study population was analysed using the systematically selected stool samples only (n = 385). Using the RLDT, the prevalence of Shigella and ETEC was 19.7% (76 of 385) and 17.7% (68 of 385) respectively. Among the 385 children, 6.2% (24) was LT-ETEC, 5.5% (21) ST-ETEC and 6.0% (23) was LT + ST-ETEC (Fig. 4).

The distribution of age groups and clinical outcomes among all diarrhea and ETEC and Shigella diarrhea are described in the supplementary table 1. The isolation rate of Shigella among the children with diarrhea in age group ˃2 years was higher (37.7%) compared to ≤ 2 years (19.4%) while the ETEC isolation rate was similar in both the age groups 14.0% in ≤ 2 and 15.9% in > 2 years old. Among the ETEC positive children, the frequency of LT-ETEC (40.4% vs 18.2%) was higher in children ≤ 2 years of age, LT + ST-ETEC (34.0% vs 54.5%) was higher in children ˃2 years while ST-ETEC was similar (25.5% vs 27.3%) in both the age groups below and over 2 years of age.

The older kids (> 2 years of age) compared to the infants (< 2 years) were more likely to develop clinical symptoms including vomiting [OR 1.93 (95% CI 1.11–3.45; p = 0.0222)], abdominal pain [OR 3.89 (2.06–7.22; p < 0.001)] and dehydration, [OR 2.66 (1.54–4.57; p < 0.001)]. Any dehydration was observed in 34.5% (20 of 58) and 50.6% (46 of 91) among ETEC and Shigella positives respectively. Among the children with ETEC positive diarrhea with dehydration, 45% was LT-ETEC, 30% ST-ETEC, and 25% was LT + ST-ETEC. Diarrhea episodes were significantly associated with fever [OR 2.32 (1.44 – 3.74, p = 0.0005)] when Shigella was detected and associated with abdominal pain [OR 2.37 (1.16 – 4.61, p = 0.0136)] when ETEC was detected. Abdominal pain was significantly associated with LT + ST-ETEC infected patients [OR 4.07 (1.55–10, p = 0.014)] compared to children with non LT + ST-ETEC diarrhea.

The frequently prescribed antibiotics at the hospitals for the treatment of diarrhea were fluoroquinolone and metronidazole, along with probiotics. Data on which participants had taken antibiotics prior to visiting the hospital was not available. Antibiotic treatment was prescribed at the hospital to 8.6% (33/385) of the children with diarrhea in the study. The odds of receiving antibiotic treatment were higher if the diarrhea episode was associated with vomiting [OR 4.37 (1.79–13; p = 0.003] and abdominal pain [OR 17.4 (8.01–39; p < 0.001)]. The proportion of children who received antibiotics among the ETEC positives (7 of 58, 12.0%) and Shigella positives (11 of 91, 12.1%) was similar. Among ETEC and Shigella positive children, the odds of getting antibiotics were significantly higher if abdominal pain was present [OR 32.2 (4.69, 65) p < 0.002)] and [OR 9.47 (2.41–39) p < 0.001] respectively. Children with ST-ETEC diarrhea had a higher odds of receiving antibiotics [OR 3.31 (1.04, 8.97; p = 0.026)] compared to non ST-ETEC diarrhea.

## Discussion

This study was the first field evaluation of RLDT for Shigella in an endemic country and the first evaluation of RLDT for ETEC in Asia. Here, we established that the performance of Shigella and ETEC RLDT are comparable to the quantitative PCR, with the sensitivity and specificity ranging from 93 to 100%, and excellent agreement between the two assays. While the RLDT assay was much more sensitive than the culture, almost all the culture positives were also positive by the RLDT. The RLDT could detect additional 12% Shigella and 13% ETEC among the samples which were culture negatives. The performance (sensitivity and specificity compared to qPCR and culture) of the RLDT in India was comparable to the previous reports of the RLDT evaluation studies at JHU^10^ and in Zambia^15^.

Shigella and ETEC are important pathogens that are largely responsible for foodborne illnesses occurring around the world^16,17^, particularly in low and middle-income countries^4^. Active and rapid monitoring through surveillance is essential for the control of these bacterial infections and outbreaks^18^. Our study showed that the RLDT is a simple and rapid gene amplification tool to detect ETEC and Shigella directly from the stool. Since the assay is simple and the hands-on time is less than five minutes, this could be implemented at the study site in Kolkata, India, with minimal training. The RLDT method is less time consuming than culture-based techniques, conventional PCR assays, and qPCR, and therefore, could be performed easily and rapidly by the staff at the study site.

ICMR-NICED under the Government of India, is one of the few institutes in India where a surveillance system for enteric diseases has been established and performed routinely. The stool specimens from this systematic surveillance system are examined at the microbiology laboratories at the NICED for common enteric pathogens using the established methods. For Shigella, culture, and ETEC, culture followed by PCR of the *E. coli* isolates are used. The surveillance data are sent to the ICMR and the Ministry of Health, India. In the previously published reports from this surveillance system, Shigella was isolated at 7.9% (51/648) from children < 5 years old in 2007 to 2009^19^ and 7.7% (193/2489) from all ages in 2001 to 2004^20^. ETEC was isolated at 4.2% (27/648) from < 5 years in 2007 to 2009^15^; from all age groups, 4.3% (164/3826) in 2008–2011^21^, and 3.7% (329/8891) in 2012–2019^22^.

While the isolation rates in this study by culture were similar to the previous reports by the ICMR-NICED^22,23^, qPCR and RLDT, had isolation rates that were much higher, ~ 3 folds for Shigella and ~ 4 folds for ETEC compared to culture. Therefore, the culture method largely underestimates the actual burden of these pathogens. Understanding the real burden of these significant pathogens at the national and sub-national levels is critical to guide global and local public health officials and policymakers to prioritize resources for accelerating vaccine development and implementation and other interventions to control these enteric infections. To strengthen surveillance of enteric diseases like Shigella and ETEC and rapid reporting at the national level to estimate disease prevalence and rapid response to contain outbreaks, a rapid, simple, as well as sensitive tool like the RLDT would be useful. In addition, rapid and simple detection of ETEC and Shigella using RLDT could guide the treatment decisions with antibiotics for these pathogens which could reduce the overuse of antibiotics at the health care facilities.

Since RLDT can be easily implemented in the endemic countries, it could be a useful screening tool in the Shigella and ETEC vaccine candidate evaluations in the upcoming phase III trials. Since the assay is rapid, the samples positive by the RLDT could be cultured and isolated colonies tested for antimicrobial resistance and whole genome sequencing.

This study has limitations. We used a stringent cutoff of ~ 10^4^ CFU/gm of stool for Shigella, which reduced the sensitivity of the RLDT compared to qPCR, which was increased when the cutoff was increased. In addition, the sample preparation methods for RLDT, qPCR, and culture were different. RLDT was done directly from the stool with minimum sample treatment; qPCR was done from purified DNA; isolates from culture were tested with biochemical test and serogrouping for Shigella and PCR for ETEC. Therefore, the sensitivity of these assays depends not only on the amplification technology but also on the starting material. Although the prevalence of EIEC is very low in this region^2^, since the *ipaH* gene is present in both Shigella and EIEC*,* it is possible that a few of the samples that are positive by the RLDT and qPCR but negative by culture for Shigella could be positive for EIEC.

In conclusion, the results of our study demonstrated that the RLDT assay is a sensitive molecular test for rapid and reliable identification of Shigella and ETEC infections from the stool. This study also showed that the RLDT could be easily implemented in an endemic setting like Kolkata, and the assay is much simple and rapid compared to qPCR, PCR, and culture methods. The RLDT is a simple and practical tool for the detection of Shigella and ETEC, which has the potential to be used for disease surveillance, outbreak detection, and vaccine evaluation in phase III trials and for on-site patient diagnosis for treatment.

## Methods

### Study design

Training of the RLDT assay: A team from the Johns Hopkins University visited the Indian Council of Medical Research (ICMR)—National Institute of Cholera and Enteric Diseases (NICED), Kolkata, and performed training on the RLDT and the qPCR for ETEC and Shigella. For the RLDT training, a written protocol and a video of the procedures were provided to the NICED staff followed by hands-on training on the first day. The staff at the NICED then performed the RLDT assay under the observation of the JHU trainers for the next 2 days.

### Sample collection and selection

In the systematic active surveillance at the NICED, stool specimens were collected from acute diarrheal patients seeking care at the Infectious Disease Hospital (IDH) and B C Roy Memorial Children hospital (BCRM) representing every fifth case with diarrhea on two randomly selected days in a week. Informed consent was obtained from each patient or parents in the case of children and enrolled in this study. Stool specimens were collected before any administration of antibiotics at the hospitals. Clinical symptoms e.g., loose/watery or bloody diarrhea, degree (severe, some or no, according to the WHO guideline)^24^ of dehydration, abdominal pain, vomiting, and fever were recorded. Stool specimens were collected in sterile McCartney bottles and transported from the study hospitals within 2 h to the microbiology laboratory of the NICED. Stool samples were tested for 24 enteric pathogens comprising bacterial, viral, and parasitic pathogens using a combination of conventional, immunological, and molecular methods at the NICED^19^ and an aliquot of the stool samples were stored at -80 °C. The clinical, demographic, and laboratory data were checked manually and entered into pre-designed data entry software (Oracle, India).

For this study, stool samples were selected from every 5^th^ patient under 5 years old children from the systematic surveillance from 2011 to 2019 (samples frozen at − 80 °C) along with the freshly collected stool samples in 2020. The total sample size of < 5 years old children was 2763 of which 1925 were stool samples and remaining 838 were rectal swab samples. For this study, every 5^th^ stool sample was selected which totaled to 385 (20%) stool samples.

To increase the number of Shigella positive samples, twenty additional stool samples which were bloody or mucoid, or both were added. The total sample size in this study was 405 (385 systematic samples + 20 suspected Shigella cases).

### Culture for Shigella and ETEC

Soon after collection, the fresh stool specimens were cultured for Shigella and ETEC, and serogroups of Shigella were identified. For detection of Shigella, stool samples were cultured on Hekton Enteric agar (HEA agar) and Xylose Lysine Deoxycholate agar* (*XLD agar*)* (DB, Difco, Sparks, MD, USA, followed by biochemical tests and serotyped using commercially available antisera (Denka Seiken, Tokyo, Japan). For detection of ETEC, stool samples were cultured on MacConkey agar (BB, Difco, Sparks, MD, USA) and three isolated *E. coli-*like lactose-fermenting colonies were tested using multiplex conventional PCR assay for the detection of LT and ST toxin genes^25^. This PCR used common primer pairs for ST which was not distinguished by STh and STp.

### Quantitative PCR and RLDT assay

DNA was extracted from 200 mg (200 μl) of stool using a bead beater followed by QIAmp Stool Mini Kit (Qiagen, Valencia, CA, USA)^10^. The concentrations of DNA were determined by measuring the optical density at 260 nm with a Nano Drop Bio Spectrometer (Eppendorf, Hamburg, Germany). The qPCR was performed with the isolated DNA to detect the target genes LT, STh, and STp for ETEC and *ipaH* for Shigella. The qPCR assay was performed using an SYBR Green master mix (Roche Diagnostics) and was carried out in a 25 μL reaction mixtures containing 1X PowerUp SYBR Green Master Mix, 0.2 μM of each primer and 2.5 μL of sample DNA and amplified for 40 cycles of 95 °C for 15 s and 60 °C for 1 min using a Roche Light-Cycler 480 (Roche Diagnostics, Penzberg, Germany)^10^. ETEC strain H10407 and *S. flexneri* 2a 2457 T were used as positive controls.

### RLDT

RLDT was conducted at the NICED directly from the stool samples using the RLDT kits to detect LT, STh, and STp genes for ETEC and *ipaH* for Shigella as described by Chakraborty et al^14^. In short, samples were added to the sample processing tube with lysis buffer followed by heat lysis. The processed samples were then added to the lyophilized ETEC and Shigella RLDT reaction strips. Each strip consisted of 8 tubes, organized as two reaction tubes each for LT, STh, and *ipaH* and one tube for STp. One reaction tube was added as the RLDT inhibitor control^14^. The strips were run for 40 min in the handheld, battery-operated, RLDT real-time fluorometer reader (Agdia Inc, IN, USA)^14^.

### Data analysis

To determine the sensitivity, specificity, PPV, NPV and accuracy of the RLDT for ETEC and Shigella the results were compared between qPCR, RLDT, and culture. The qPCR and RLDT tests were run and interpreted by two lab personnel blinded to each other. “ETEC total” was considered positive when at least one of the ETEC genes, LT, STh, or STp was positive. The sensitivity and specificity values were expressed as percentages. For analysis, the cut-off value of qPCR to determine positives was assigned as the lowest detection limits (LOD) of the RLDT assays, 10^4^ CFU/gm of stool (corresponds to qPCR Cq of 30.49) for Shigella and 10^5^ CFU/gm of stool for ETEC genes (corresponds to qPCR Cq of 28.2, 28.6 and 30.07 for LT, STh, and STp respectively)^10^. The agreement between two assays were detected using Cohen’s Kappa. Cohen's kappa does not require the specification of a gold standard but simply quantifies the agreement between binary outcomes of tests (positive or negative), considering agreement occurring by chance^26,27^. The following labels were assigned to the corresponding ranges of kappa strength: poor agreement, < 0; slight, 0.0 to 0.20; fair, 0.21 to 0.40; moderate, 0.41 to 0.60; substantial, 0.61 to 0.80; and almost perfect, 0.81 to 1.00. The Statistical analyses were performed, and the figures were prepared using Stata Corp LLS (Version 16) and GraphPad, CA (Version 9). Simple logistic regression models were built to evaluate the association between age group, clinical symptoms, antibiotic use, and diarrhea pathogens. The analyses were performed with R version 4.1.2 (R Foundation for Statistical Computing, Vienna, Austria).

### Ethics statement

The study was reviewed and approved by the institutional Ethical Review Committee at the ICMR- NICED and the Institutional Review Board of the Johns Hopkins Bloomberg School of Public Health. Written informed consent was obtained from the parents / legal guardians of the children. All methods were performed in accordance with the relevant guidelines and regulations.

### Supplementary Information

Supplementary Table 1.

## Data Availability

All data generated or analysed during this study are included in this manuscript and its Supplementary Information file.
