# Supplementary material for: Field evaluation of a simple and rapid diagnostic test, RLDT to detect Shigella and enterotoxigenic E. coli in Indian children
Source: Sci Rep. 2024 Apr 16;14:8816. doi: 10.1038/s41598-024-59181-6 (PMC11021469; doi:10.1038/s41598-024-59181-6)
Supplement: Supplementary file 1 — Supplementary Table 1. [file 41598_2024_59181_MOESM1_ESM.docx]

**Supplementary Table 1. Distributions of the age groups and clinical outcomes.**

|  | **All diarrhea**  **N=405** | **ETEC**  **N=58** | **Shigella**  **N=91** |
| --- | --- | --- | --- |
| **Age Group n (%)** |  |  |  |
| 0-1 | 192 (47.4%) | 23 (39.7%) | 22 (24.2%) |
| >1-2 | 144 (35.6%) | 24 (41.4%) | 43 (47.3%) |
| >2-5 | 64 (15.8%) | 10 (17.2%) | 23 (25.3%) |
| >5 | 5 (1.2%) | 1 (1.7%) | 3 (3.3%) |
| **Diarrhea type n (%)** |  |  |  |
| Watery | 327 (80.7%) | 47 (81.0%) | 66 (72.5%) |
| Loose | 37 (9.1%) | 8 (13.8%) | 8 (8.8%) |
| Bloody | 1 (0.2%) | 0 (0.0%) | 1 (1.1%) |
| Mucoid | 21 (5.2%) | 2 (3.4%) | 5 (5.5%) |
| Bloody & Mucoid | 19 (4.7%) | 1 (1.7%) | 11 (12.1%) |
| **Dehydration Status n (%)** |  |  |  |
| Severe | 4 (1.0%) | 2 (3.4%) | 2 (2.2%) |
| Some | 162 (40.0%) | 18 (31.0%) | 44 (48.4%) |
| No | 239 (59.0%) | 38 (65.5%) | 45 (49.5%) |
| **Vomiting n (%)** |  |  |  |
| Yes | 237 (58.5%) | 37 (63.8%) | 56 (61.5%) |
| No | 168 (41.5%) | 21 (36.2%) | 35 (38.5%) |
| **Fever %** |  |  |  |
| Yes | 146 (36.0%) | 14 (24.1%) | 47 (51.6%) |
| No | 259 (64.0%) | 44 (75.9%) | 44 (48.4%) |
| **Abdominal Pain n (%)** |  |  |  |
| Yes | 55 (13.6%) | 14 (24.1%) | 15 (16.5%) |
| No | 350 (86.4%) | 44 (75.9%) | 76 (83.5%) |
